# Genomic Selection for Economic Traits in Inner Mongolia Cashmere Goats by Integrating GWAS Prior Information

**DOI:** 10.3390/vetsci12100996

**Published:** 2025-10-15

**Authors:** Haijiao Xi, Qi Xu, Huanfeng Yao, Zihao Shen, Bohan Zhou, Qi Lv, Jinquan Li, Ruijun Wang, Yanjun Zhang, Rui Su, Zhiying Wang

**Affiliations:** 1 College of Animal Science, Inner Mongolia Agricultural University, Hohhot 010018, China; xihaijiao456@163.com (H.X.); 13015206915@163.com (Q.X.); 15661397936@163.com (H.Y.); lyf2022202010010@126.com (Z.S.); bohanzhou123456@163.com (B.Z.); lvqi1202@imau.edu.cn (Q.L.); 13284731747@163.com (J.L.); nmgwrj@126.com (R.W.); imauzyj@163.com (Y.Z.); suruiyu@126.com (R.S.); 2 Key Laboratory of Goat and Sheep Genetics, Breeding and Reproduction in Inner Mongolia Autonomous Region, Hohhot 010018, China; 3 Key Laboratory of Mutton Sheep and Goat Genetics and Breeding, Ministry of Agriculture, Hohhot 010018, China

**Keywords:** Inner Mongolia Cashmere goats, genomic selection, GWAS prior marker information, dominance effects

## Abstract

The accuracy of genomic selection has a significant impact on the selection of superior individuals in livestock. Studies have reported that integrating GWAS information can improve the accuracy of genomic prediction. In this study, phenotypic data, systematic environmental data, and genotypic data of important economic traits (cashmere yield, cashmere diameter, body weight, and cashmere length) of Inner Mongolia cashmere goats were utilized. Based on the results of a previous genome-wide association study that considered additive and dominance effects, the top 5%, top 10%, top 15%, and top 20% of loci were extracted as prior marker information. The genomic breeding values for each trait were estimated using the GBLUP–GA method based on GWAS prior information, and the accuracy of genomic prediction was further evaluated using a five-fold cross-validation method. The results showed that the contribution of significant loci to the genetic variance of each trait gradually increased with an increase of the number of integrated loci. The genetic variance contribution rates of significant loci to cashmere yield, cashmere diameter, body weight, and cashmere length were 64–71%, 47–57%, 76–82%, and 66–80%, respectively. The additive heritability estimates for cashmere yield, cashmere diameter, body weight, and cashmere length using GWAS prior information were 0.252–0.266, 0.297–0.580, 0.305–0.330, and 0.107–0.117, respectively. These values were higher than those obtained using the traditional G matrix constructed from all loci, with increases of 0.052–0.066, 0.007–0.29, 0.134–0.159, and 0.015–0.025, respectively. The results of genomic prediction accuracy showed that when 5% of the GWAS prior information was integrated, the highest genomic prediction accuracy was achieved for cashmere yield (0.8156), body weight (0.8361), and cashmere length (0.7571). When 20% of the GWAS prior information was integrated, the genomic prediction accuracy for cashmere diameter was 0.8074, which was significantly higher than that at other levels. Additionally, it was found that the dominance heritability for cashmere diameter, body weight, and cashmere length was very small and could be ignored when integrating GWAS prior information. Therefore, when integrating prior information for genomic selection of these traits, the influence of dominance effects can be disregarded.

## Simple Summary

This study accelerated the genetic improvement of Inner Mongolia cashmere goats by integrating functional biological information. Additionally, it discussed the influence of dominance effects on the accuracy of genomic selection for the economic traits in Inner Mongolia cashmere goats. The aim was to accurately select superior individuals and enhance the industrial economic benefits of cashmere goats.

## 1. Introduction

The Inner Mongolia cashmere goats (IMCGs) are a dual-purpose breed for both cashmere and meat production. The cashmere fiber sourced from Inner Mongolia exhibit exceptional length, fineness, silky softness, and natural luster, meeting the stringent quality standards of luxury textile manufacturing. Renowned as a premium raw material in global textile supply chains, this cashmere contributes substantially to the regional economy, generating an annual output value and driving the development of livestock and high-end textile industries in the Inner Mongolia Autonomous Region [1]. Moreover, the meat of Inner Mongolia cashmere goats is rich in various nutrients, including proteins, vitamins, minerals, and possesses high nutritional value and health-promoting properties, making it an excellent source of high-quality animal protein [2]. Genomic selection (GS) considering non-additive genetic effects (dominance and epistasis) has been widely applied in genetic evaluation of various livestock species, including cattle [3,4], sheep [5,6], and pigs [7,8]. Bhuiyan et al. [9] developed five animal models based on genotype data from Hanwoo cattle, considering different combinations of additive, dominance, and epistatic effects, as well as their interactions, which evaluated the accuracy of genomic breeding value predictions for carcass and meat quality traits. It was found that the animal model incorporating both dominance and epistatic effects can significantly improve the accuracy of genomic breeding values for these traits in Hanwoo cattle. Sadeghi et al. [10] conducted genetic evaluations of growth traits in Adani goats. It was found that the animal model incorporating dominance and epistatic effects improve the accuracy of estimated breeding values for growth traits. Mei et al. [11] estimated the breeding value accuracy for growth traits in Yorkshire pigs using genotyping data from 55 K and 42 K SNP chips. It was found that the model including dominance effects can enhance the prediction accuracy for litter size.

The results of GWAS have been widely regarded as an important type of biological prior information of genomic selection, providing valuable insights for the optimization of GS models [12,13,14]. The *p*-values obtained from GWAS can indicate the magnitude of the genetic contribution of each SNP to a particular trait, with smaller *p*-values suggesting a more significant genetic contribution from the SNP. Numerous studies have demonstrated that integrating GWAS prior information into GS models can significantly enhance the accuracy of genomic prediction [15,16,17]. However, previous genomic selection studies in cashmere goats have assumed that all SNPs contribute equally to the phenotype, without considering the unique contributions of significant loci. This approach overlooks the biological importance of GWAS-significant loci, which to some extent restricts the model’s ability to parse key functional variations and consequently affects the precision of genomic selection. Therefore, it is necessary to conduct research that effectively integrates the biological information of GWAS significant loci into the genomic selection models for economic traits in cashmere goats, with the expectation of improving the accuracy of genomic prediction for these traits.

Based on the previous GWAS results of important economic traits in IMCGs analyzed by our research group using both additive and dominance effects, this study selected the most significant 5%, 10%, 15%, and 20% SNPs as prior marker information according to the *p*-values, with the remaining SNPs serving as residual marker information. The genetic variance contributions of the prior and residual marker information were calculated separately to determine the weight coefficients of the relationship matrix. Subsequently, the genomic breeding values were estimated under different proportions of prior marker information using the GBLUP–GA method based on the integrated genomic structure matrix. Finally, the accuracy of genomic breeding value estimation obtained without using marker information and with different proportions of marker information was evaluated using a five-fold cross validation method to comprehensively assess the application effect of integrating GWAS prior marker information in genomic selection of economic traits in IMCGs.

## 2. Materials and Methods

### 2.1. Source of Experimental Animals

The data used in this study were derived from the production performance records of 2299 individuals from Inner Mongolia Yiwei White Cashmere Goat Co., Ltd., Ordos, China. The traits included cashmere yield (CY), cashmere diameter (CD), body weight (BW), and cashmere length (CL), with relevant records being reliable and detailed.

### 2.2. Sources of Phenotypic and Genotypic Data

The phenotypic data used in this study were the records of economic traits of IMCGs accumulated by our research group previously. The number of records for cashmere yield, cashmere diameter, body weight, and cashmere length were 9027, 5342, 8549, and 9601, respectively, with corresponding mean phenotypic values of 772.05 g, 14.95 um, 37.20 kg, and 6.17 cm. The standard deviations were 228.73, 0.81, 7.68, and 1.08, and the coefficients of variation were 29.60%, 5.40%, 21.04%, and 17.50%, respectively. The genotypic data were derived by genotyping the goat 70 K SNP chip. A total of 67,088 raw SNPs were initially obtained, and after quality control, 50,728 SNPs were retained for subsequent analyses. For more details, refer to the previous report by Xu [18].

### 2.3. Model for Genomic Selection by Integrating Prior GWAS Information

Based on the phenotypic data, systematic environmental data, and genotypic data of economic traits in IMCGs, a mixed animal model was established to perform the genomic selection in this study. The fixed effects included flock, age of individual, sex, and years of measurement [19]. The random effects included the additive genetic effects, dominance genetic effects, permanent environmental effects of individual, and residual effects. The GBLUP–GA method incorporating GWAS prior information was subsequently utilized to estimate variance components and genomic breeding values. The model equation is presented as follows:

y = Xb + Zu + Wp + Vd + e

where y is the vector of phenotype in each trait; b is the vector of fixed effects; u is the vector of individual additive genetic effects, u~N(0, *G*$\sigma_{u}^{2}$), and G is the genomic relationship matrix; p is the vector of individual permanent environmental effects; d is the vector of individual dominance genetic effects; X, Z, W, and V are the incidence matrices for the effects in b, u, p, and d, respectively; e is the residual effects of the trait. All statistical analyses were performed using the ASREML (4.2) software package. Model convergence was successfully achieved for all analyses (ASREML convergence criterion: TRUE), indicating stable and reliable parameter estimates.

GBLUP method is Genomic Best Linear Unbiased Prediction. It can be used to predict the breeding ability of individuals using genomic data, environmental effects, and phenotypic values. The corresponding equations are as follows:

$$\left[\begin{array}{cccc} X\prime X & X\prime Z & X\prime W & X\prime V\\ Z\prime X & Z\prime Z+\alpha_{1}G^{-1} & Z\prime W & Z\prime V\\ W\prime X & W\prime Z & W\prime W+I\alpha_{2} & W\prime V\\ V\prime X & V\prime Z & V\prime W & V\prime V+\alpha_{3}D^{-1} \end{array}\right]\left[\begin{array}{c} \hat{b}\\ \hat{u}\\ \hat{p}\\ \hat{d} \end{array}\right]=\;\left[\begin{array}{c} X\prime y\\ Z\prime y\\ W\prime y\\ V\prime y \end{array}\right]$$

In GBLUP, G is the matrix relating to additive genetic effects for the genomic relationship matrix (G $=\frac{ZZ\prime }{2\sum_{pi}\left(1-pi\right)}$, D = $\frac{VV\prime }{4\sum {pi}^{2}{qi}^{2}}$).

The additive (G) [20] and dominance (D) [21] genomic relationship matrices were constructed according to established methods. For the additive relationship matrix (G), the elements of matrix M were coded as {0, 1, 2} corresponding to the number of alternative allele copies. This matrix was then standardized by adjusting for allele frequencies using the transformation Z = M − P, where P is a matrix where each column contains the value 2pi (twice the allele frequency at locus i). For the dominance matrix, the elements of matrix V were coded as 0 for homozygous genotypes and 1 for heterozygous genotypes, which was also centered.

The introduction of the GBLUP–GA method used in this study was as follows. Based on the *p*-values of loci from GWAS results considering additive and dominance effects, the loci were sorted according to their *p*-values from smallest to largest. The top 5%, top 10%, top 15%, and top 20% of loci were extracted as a set of prior marker information to construct the relationship matrix. The remaining loci were used to construct another relationship matrix. Variance components and genetic parameters were estimated using these two matrices, yielding additive genetic variances $\sigma_{Ga1}^{2}$ and $\sigma_{Ga2}^{2}$ and dominance genetic variances $\sigma_{Gd1}^{2}$ and $\sigma_{Gd2}^{2}$. The contribution rate of the prior marker information to the genetic variance was calculated as *τ=*$\frac{\sigma_{Ga1}^{2}+\sigma_{Gd1}^{2}}{\sigma_{Ga1}^{2}+\sigma_{Gd1}^{2}+\sigma_{Ga2}^{2}+\sigma_{Gd2}^{2}}$ Subsequently, the relationship matrix was fitted using the proportion of genetic variance explained as the weight, with the formula given by $G_{t}$:

$$G_{t}=\tau G_{1}+(1-\tau )G_{2}$$

$G_{1}$ represents the relationship matrix based on prior marker information; $G_{2}$ represents the relationship matrix based on the remaining loci; τ represents the contribution rate of prior marker information to the genetic variance of the trait.

### 2.4. Evaluation for Accuracy of Genomic Breeding Value

In this study, we employed a five-fold cross validation method to evaluate the accuracy of genomic breeding values of economic traits in IMCGs. Additionally, we constructed a generalized linear model with the accuracy of breeding values obtained under each prior marker information scenario as the dependent variable and the proportion of marker information as the independent variable. The ANOVA function [22] in the R language was used to analyze the impact of integrating prior information on the accuracy of genomic prediction for economic traits in IMCGs. Moreover, the significance of differences in genomic prediction accuracy under various prior information scenarios using GraphPad Prism (9.5) [23] were visualized.

The individuals were divided into five groups, and then one group was selected as the validation population at each time, and the other four groups were used as the training population. Five groups of individuals will be used as the validation population in turn. The accuracy of genomic predictions was assessed by dividing the covariance of the adjusted phenotypic values and estimated breeding values (*cov*(*a.p*)) by the square of heritability (*h*^2^).

$$r=\frac{cov(a,p)}{\sqrt{h^{2}}}$$

## 3. Results

### 3.1. Statistics of Significant SNPs Based on Different Prior Marker Information

**Figure 1:**
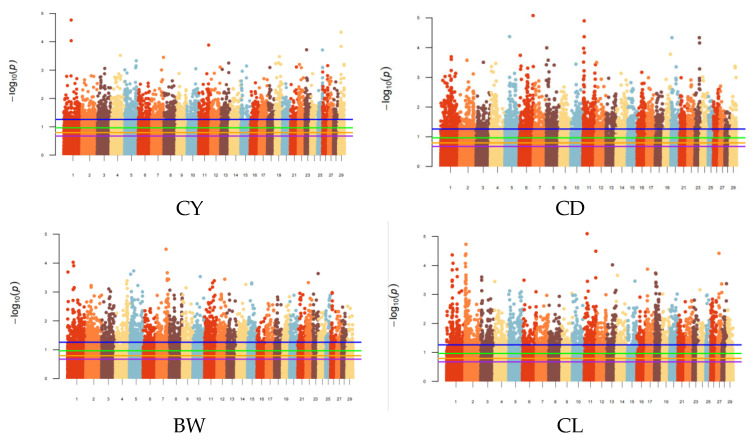
Statistics of significant SNPs under different prior marker information scenarios.

Based on the results of the GWAS using the additive-dominance genetic effect model, significant SNPs were identified by setting the top 5%, top 10%, top 15%, and top 20% according to *p*-values from smallest to largest. The results are shown in Figure 1. The number of statistically significant SNPs demonstrated a progressive increase in correlation with elevated threshold levels There were 2536, 5073, 7609, and 10,145 significant SNPs under the top 5%, top 10%, top 15%, and top 20% thresholds, respectively, and these SNPs were evenly distributed across all chromosomes.

### 3.2. Estimation of Variance Components and Genetic Parameters for Economic Traits Based on Prior Marker Information

#### 3.2.1. Estimation of Variance Components and Genetic Parameters for Cashmere Yield Based on Prior Marker Information

Based on the GWAS results, the G matrix was optimized by extracting the top 5%, top 10%, top 15%, and top 20% SNPs to estimate the genetic parameters for CY in IMCGs using the GBLUP–GA method. The results are shown in Table 1. The additive heritability for CY ranged from 0.252 to 0.266, while the dominance heritability ranged from 0.053 to 0.062; the repeatability for CY ranged from 0.305 to 0.329, indicating that CY is a trait with moderate to high heritability. The top 5% to top 20% of SNPs had a substantial impact on CY, accounting for 64% to 71% of the total genetic variance. The additive genetic variance for CY were 9672.55 to 10,704.93. dominance genetic variance for CY were 2049.59 to 2511.09. The contribution of permanent environmental effects to the phenotype was relatively small, ranging from 3.37 × 10^−7^ to 4.40 × 10^−7^.

#### 3.2.2. Estimation of Variance Components and Genetic Parameters for Cashmere Diameter Based on Prior Marker Information

Based on the GWAS results, the G matrix was optimized by extracting the top 5%, top 10%, top 15%, and top 20% SNPs to estimate the genetic parameters for CD in IMCGs using the GBLUP–GA method. The results are shown in Table 2. The additive heritability for CD ranged from 0.297 to 0.580, while the dominance heritability ranged from 2.06 × 10^−7^ to 8.46 × 10^−7^; the repeatability for CD ranged from 0.297 to 0.581, indicating that CD is a trait with high heritability. The top 5% to top 20% of SNPs had a relatively smaller impact on CD, accounting for 47% to 57% of the total genetic variance. The contribution of permanent environmental effects to the phenotype was relatively small, ranging from 4.71 × 10^−8^ to 1.30 × 10^−5^. The additive genetic variance for CD were 0.161 to 0.316. dominance genetic variance for CD were 1.02 × 10^−7^ to 4.60 × 10^−7^.

#### 3.2.3. Estimation of Variance Components and Genetic Parameters for Body Weight Based on Prior Marker Information

Based on the GWAS results, the G matrix was optimized by extracting the top 5%, top 10%, top 15%, and top 20% SNPs to estimate the genetic parameters for BW in IMCGs using the GBLUP–GA method. The results are shown in Table 3. The additive heritability for BW ranged from 0.305 to 0.330, while the dominance heritability ranged from 1.11 × 10^−8^ to 6.77 × 10^−8^; the repeatability for BW ranged from 0.414 to 0.431, indicating that BW is a trait with high heritability. The top 5% to top 20% of SNPs had a substantial impact on BW, accounting for 76% to 82% of the total genetic variance. The contribution of permanent environmental effects to the phenotype was small, ranging from 2.83 to 3.23. The additive genetic variance for BW were 8.96 to 9.90. dominance genetic variance for BW were 1.17 × 10^−7^ to 6.31 × 10^−7^.

#### 3.2.4. Estimation of Variance Components and Genetic Parameters for Cashmere Length Based on Prior Marker Information

Based on the GWAS results, the G matrix was optimized by extracting the top 5%, top 10%, top 15%, and top 20% SNPs to estimate the genetic parameters for CL in IMCGs using the GBLUP–GA method. The results are shown in Table 4. The additive heritability for CL ranged from 0.107 to 0.117; the repeatability for CL ranged from 0.105 to 0.112, indicating that CL is a trait with moderate to low heritability. The top 5% to top 20% of SNPs had a substantial impact on CL, accounting for 66% to 80% of the total genetic variance. The contribution of permanent environmental effects to the phenotype was relatively small, ranging from 3.79 × 10^−6^ to 8.67 × 10^−6^. The additive genetic variance for CL were 0.112 to 0.123.

### 3.3. Evaluation of Genomic Prediction Accuracy for Economic Traits Based on Prior Marker Information

**Figure 2:**
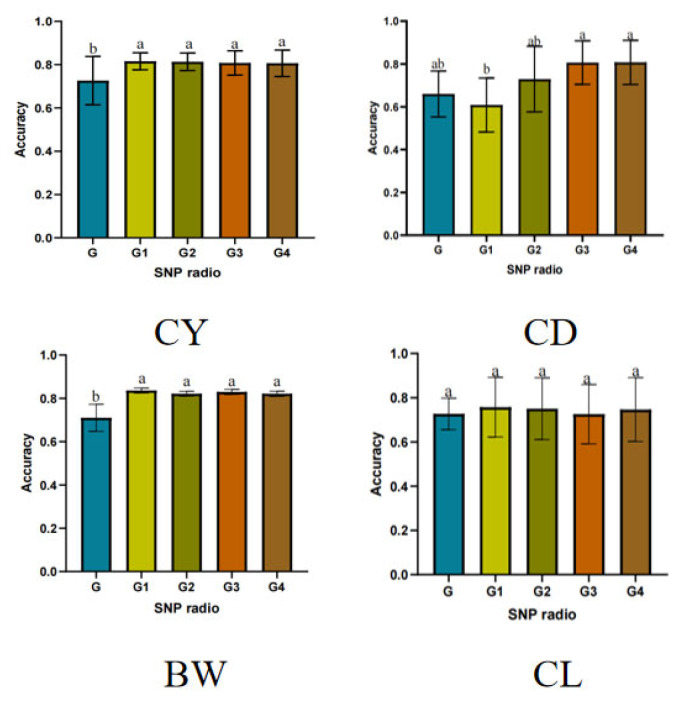
Impact of different prior marker information on the accuracy of genomic prediction for economic traits. Notes: different letters above bars indicate significant differences among groups (*p* < 0.05).

The prediction accuracy of genomic estimated breeding value for economic traits was evaluated by integrating top 5%, top 10%, top 15%, and top 20% of SNPs as prior marker information using the GBLUP–GA method. The results are shown in Tables S1–S4 and Figure 2. Integration of the top 5% SNPs as prior information significantly enhanced the prediction accuracy of GEBVs for CY, CL and BW compared to the scenario without prior information. No significant differences were observed in prediction accuracy when integrating the top 5%, 10%, 15%, or 20% of SNPs. However, the use of the top 20% SNPs significantly improved the prediction accuracy for CD compared to both the top 5% SNP set and the model without prior information. For CY, BW, and CL, the highest genomic prediction accuracy with the GBLUP–GA method was achieved by integrating the top 5% SNPs, with accuracies of 0.8156, 0.8361, and 0.7571, respectively. For CD, the highest genomic prediction accuracy was achieved by integrating 20% of the loci using the GBLUP–GA method, the value is 0.8074.

## 4. Discussion

In this study, the top 5%, top 10%, top 15%, and top 20% SNPs were selected as prior marker information by ranking the *p*-values from GWAS results, and they were effectively integrated into the animal model to enhance the prediction accuracy of genomic breeding values in livestock. Juan et al. [24] fitted five models for genomic selection in Holstein bulls, including the 5′ region, 3′ region, nonsynonymous region, nonsense region, and noncoding RNA. The significant differences among different models were observed, indicating that SNPs in different functional regions have different genetic contributions to the traits. Gao et al. [25] used GBLUP method with haplotypes or SNP to conduct genomic selection studies in three different populations: rice, Arabidopsis, and yellow-feathered chickens. They discovered that the genomic prediction accuracy varied among different models and species. Ni et al. [26] constructed different weighted genomic relationship matrices based on whole-genome sequencing data in chickens. The study revealed that the breeding values for egg production traits achieved the highest accuracy when the unweighted genomic relationship matrix was utilized. Song et al. [27] evaluated the prediction accuracy of genomic breeding values for growth traits in aquatic animals using BLUP, GBLUP, BayesR, WGBLUP, and GFBLUP methods. It was found that the average accuracy of GEBV using the WGBLUP method was higher than that using the GBLUP method. These studies have revealed the role of model optimization from the perspectives of functional genomics and SNP weight allocation. Therefore, it is necessary to assign different weights to different SNPs in genome selection of livestock.

The additive heritability for CY, CD, BW, and CL based on GWAS prior information ranged from 0.252 to 0.266, 0.297 to 0.580, 0.305 to 0.330, and 0.107 to 0.117, respectively. These values were higher than those obtained using the original G matrix by 0.052 to 0.066, 0.007 to 0.29, 0.134 to 0.159, and 0.015 to 0.025, respectively. In this study, we used the results of GWAS as prior information to construct the genomic relationship matrix, which is believed to yield higher heritability estimates [28]. Wang et al. [19] estimated genetic parameters in IMCGs and reported heritability values for CD, CY, and CL of 0.27, 0.24, and 0.14, respectively. These values for CD and CY were slightly lower than those found in our study. Kibuuka et al. [29] used ASREML software to construct a mixed animal model to estimate genetic parameters for growth traits in Tswana goats. They reported a heritability of 0.48 for BW, which was higher than the value obtained in our study. Li et al. [30] estimated genetic parameters for cashmere traits in Alpine Merino sheep and reported a heritability of 0.20 for average fiber diameter. Ramos et al. [31] conducted a genetic evaluation of wool traits in Uruguayan Merino sheep and reported heritability estimates of 0.73 for fiber diameter in yearlings and 0.71 for fiber diameter in adult ewes. Ahmad et al. [32] estimated genetic parameters for various fiber traits in Rambouillet sheep using a multi-trait animal model and reported additive heritabilities of 0.120, 0.136, and 0.356 for greasy fleece weight, staple length, and fiber diameter, respectively. This discrepancy may be attributed to differences in population size and breed. The estimates of dominance heritability for each trait were lower than those obtained using the traditional G matrix [33], which may be due to the different proportions of GWAS information incorporated, the statistical power of the GWAS analysis, and the smaller sample size. Additionally, dominance genetic heritability may be influenced by various factors, such as gene interactions and environmental effects. The permanent environmental variance estimates are near zero for some traits, which may be due to a combination of uniform management practices, limited data availability, and the inherent high environmental sensitivity of the traits themselves.

In this study, the top 5% to top 20% SNPs based on *p*-values from GWAS were selected as prior information to weight the G matrix in the GBLUP model and further estimated the genomic prediction accuracy for various traits. We found that the estimated breeding values for CY, CD, BW, and CL were improved under different proportions of prior information, with increases of 11.06% to 12.31%, 10.55% to 22.39%, 15.68% to 17.78%, and 2.65% to 4.12%, respectively. In livestock breeding, previous studies have shown that integrating biological prior information can improve the accuracy of breeding value estimation for economic traits in livestock. Cai et al. [16] evaluated the accuracy of GEBV for carcass traits in Yorkshire pigs by integrating biological prior information. It found that the GFBLUP and BLUP|GA methods improved accuracy of GEBV by increases of 6.18% and 5.53%, respectively, compared to the whole SNP-GBLUP method. Zhang et al. [34] categorized imputed whole-genome resequencing data into distinct genomic regions, including introns, intergenic areas, and non-coding sequences. Utilizing the GFBLUP and GBLUP methods, they estimated GEBV for growth traits in pigs. Their study revealed that incorporating annotation information into the GFBLUP model enhanced the accuracy of GEBV prediction by 2.82%. Liu et al. [35] used the GBLUP method to integrate significant SNPs as prior information for estimating GEBV of loin muscle area in Duroc × Landrace × Yorkshire crossbred pigs. It was found that prediction accuracy was improved by 4.8%. Wang et al. [36] evaluated the accuracy of GEBV for weaning weight in plateau Merino sheep by integrating GWAS prior marker information. It showed that incorporating the top 5% to 20% SNPs from GWAS results as prior information into genomic selection models can improve the prediction ability of GEBV. Hossein et al. [37] used the ssGBLUP and WssGBLUP methods to predict the accuracy of GEBV for carcass traits in Hanwoo cattle. The results indicated that the WssGBLUP method enhanced the accuracy of GEBV for carcass weight, loin muscle area, and yearling weight by 22%, 15%, and 20%, respectively, relative to the ssGBLUP method. Heras-Saldana et al. [38] evaluated the accuracy of GEBV for carcass traits in Hanwoo cattle by integrating significant SNPs as prior information using the GBLUP and BayesR methods. It was found that the accuracy of GEBV prediction for backfat thickness and carcass weight was improved by 0.06 and 0.04, respectively. Compared to GBLUP method, the accuracy of GEBV prediction for loin muscle area with BayesR method was improved by 0.02. These studies collectively demonstrate that integrating GWAS prior information, functional annotation results [39,40], and gene expression data [41,42,43] can improve the prediction accuracy of GEBV. This study found that for CY, BW, and CL, incorporating the top 5% of genomic markers achieved the highest predictive accuracy. In contrast, for CD, integrating the top 20% of markers yielded the optimal prediction accuracy. CD is likely governed by a large number of loci with small effects. Although including only the top 5% most significant markers captures a considerable proportion of the heritability, it may still omit numerous loci scattered throughout the genome that, despite having small effects, contribute meaningfully to the trait. In comparison, other traits with lower heritability may have a genetic architecture where hereditary variation is concentrated in a relatively limited number of loci with moderate to large effects. Therefore, including only the top 5% of markers is sufficient to effectively capture the majority of their predictable genetic variance.

## 5. Conclusions

In this study, we integrated GWAS prior information to evaluate the accuracy of genomic prediction for economic traits in IMCGs. For CY, BW, and CL, the highest genomic prediction accuracy with the GBLUP–GA method was achieved by integrating the top 5% SNPs, with accuracies of 0.8156, 0.8361, and 0.7571, respectively. For CD, the highest genomic prediction accuracy was achieved by integrating 20% of the loci using the GBLUP–GA method; the value is 0.8074. Additionally, it was found that the dominance effects did not need to be considered in animal model when integrating GWAS prior information for genomic selection of CD, BW, and CL traits. These findings offer crucial methodologies for genomic prediction of economic traits in Inner Mongolia cashmere goats. Integrating GWAS information significantly improves the accuracy of genomic prediction for economic traits in Inner Mongolia cashmere goats.

## Figures and Tables

**Table 1:** Estimates of CY variance components and genetic parameters based on the GBLUP–GA method under different top SNPs proportions.

Prior Marker Information	Matrix	$\sigma_{a}^{2}$	$\sigma_{d}^{2}$	$\sigma_{p}^{2}$	$\sigma_{e}^{2}$	$h_{a}^{2}$	$h_{d}^{2}$	r	τ
Top 5%	G	7684.90	2679.40	2639.60	25,396.30	0.200 ± 0.03	0.070 ± 0.01	0.339	
	G1	9064.44	1943.28	9.88 × 10^−4^	26,416.63	0.242 ± 0.05	0.052 ± 0.01	0.294	0.64
	G2	6324.64	1.02 × 10^−3^	7542.55	27,746.20	0.152 ± 0.02	2.45 × 10^−8^ ± 0.00	0.333	0.36
	Gt	9672.55	2049.59	4.40 × 10^−7^	26,699.68	0.252 ± 0.04	0.053 ± 0.01	0.305	
Top 10%	G1	9670.59	1893.72	9.93 × 10^−4^	26,450.71	0.254 ± 0.04	0.050 ± 0.01	0.304	0.68
	G2	5490.67	1.77 × 10^−3^	8298.26	27,730.98	0.132 ± 0.02	4.26 × 10^−8^ ± 0.00	0.332	0.32
	Gt	10,288.58	2062.71	3.44 × 10^−7^	26,781.20	0.263 ± 0.03	0.053 ± 0.01	0.316	
Top 15%	G1	9929.96	2130.17	4.51 × 10^−4^	26,562.58	0.257 ± 0.04	0.055 ± 0.01	0.312	0.70
	G2	5049.34	1.20 × 10^−3^	8714.02	27,722.05	0.122 ± 0.01	2.89 × 10^−8^ ± 0.00	0.332	0.30
	Gt	10,453.66	2356.57	3.37 × 10^−7^	26,885.58	0.263 ± 0.03	0.059 ± 0.02	0.323	
Top 20%	G1	10,324.52	2208.65	4.93 × 10^−4^	26,710.11	0.263 ± 0.03	0.056 ± 0.02	0.319	0.71
	G2	5049.34	8.44 × 10^−4^	8714.02	27,722.05	0.121 ± 0.02	2.03 × 10^−8^ ± 0.00	0.332	0.29
	Gt	10,704.93	2511.09	4.03 × 10^−7^	27,012.14	0.266 ± 0.04	0.062 ± 0.02	0.329	

Notes: $\sigma_{a}^{2}$: additive variance; $\sigma_{d}^{2}$: dominance variance; $\sigma_{p}^{2}$: permanent environmental variance; $\sigma_{e}^{2}$: residual variance; $h_{a}^{2}$: additive heritability; $h_{d}^{2}$ dominance heritability; r: repeatability.

**Table 2:** Estimates of CD variance components and genetic parameters based on the GBLUP–GA method under different top SNPs proportions.

Prior Marker Information	Matrix	$\sigma_{a}^{2}$	$\sigma_{d}^{2}$	$\sigma_{p}^{2}$	$\sigma_{e}^{2}$	$h_{a}^{2}$	$h_{d}^{2}$	r	τ
Top 5%	G	0.145	0.013	0.027	0.317	0.290 ± 0.03	0.026 ± 0.01	0.368	
	G1	0.145	1.98 × 10^−7^	5.33 × 10^−7^	0.309	0.319 ± 0.05	4.37 × 10^−7^ ± 0.00	0.319	0.47
	G2	0.124	0.038	0.036	0.338	0.231 ± 0.03	0.071 ± 0.02	0.369	0.53
	Gt	0.161	3.87 × 10^−7^	4.71 × 10^−8^	0.381	0.297 ± 0.02	7.14 × 10^−7^ ± 0.00	0.297	
Top 10%	G1	0.153	1.00 × 10^−7^	1.99 × 10^−7^	0.303	0.336 ± 0.04	2.19 × 10^−7^ ± 0.00	0.336	0.51
	G2	0.107	0.038	0.051	0.338	0.200 ± 0.01	0.071 ± 0.01	0.367	0.49
	Gt	0.316	4.60 × 10^−7^	1.30 × 10^−5^	0.228	0.580 ± 0.06	8.46 × 10^−7^ ± 0.00	0.581	
Top 15%	G1	0.161	1.02 × 10^−7^	1.03 × 10^−7^	0.303	0.346 ± 0.03	2.20 × 10^−7^ ± 0.01	0.347	0.55
	G2	0.094	0.040	0.060	0.338	0.177 ± 0.02	0.075 ± 0.02	0.365	0.45
	Gt	0.181	1.02 × 10^−7^	3.60 × 10^−7^	0.313	0.362 ± 0.03	2.06 × 10^−7^ ± 0.00	0.366	
Top 20%	G1	0.167	1.95 × 10^−7^	4.87 × 10^−7^	0.304	0.354 ± 0.04	4.14 × 10^−7^ ± 0.00	0.355	0.57
	G2	0.086	0.039	0.068	0.338	0.162 ± 0.01	0.073 ± 0.03	0.363	0.43
	Gt	0.187	1.70 × 10^−7^	3.73 × 10^−7^	0.315	0.367 ± 0.03	3.39 × 10^−7^ ± 0.00	0.373	

Notes: $\sigma_{a}^{2}$: additive variance; $\sigma_{d}^{2}$: dominance variance; $\sigma_{p}^{2}$: permanent environmental variance; $\sigma_{e}^{2}$: residual variance; $h_{a}^{2}$: additive heritability; $h_{d}^{2}$ dominance heritability; r: repeatability.

**Table 3:** Estimates of BW variance components and genetic parameters based on the GBLUP–GA method under different top SNP proportions.

Prior Marker Information	Matrix	$\sigma_{a}^{2}$	$\sigma_{d}^{2}$	$\sigma_{p}^{2}$	$\sigma_{e}^{2}$	$h_{a}^{2}$	$h_{d}^{2}$	r	τ
Top 5%	G	3.13	0.16	4.14	10.82	0.171 ± 0.01	0.009 ± 0.01	0.407	
	G1	8.66	0.02	3.18	17.27	0.297 ± 0.03	6.85 × 10^−4^ ± 0.00	0.407	0.76
	G2	2.38	0.35	10.31	17.12	0.079 ± 0.01	0.012 ± 0.01	0.432	0.24
	Gt	8.96	1.99 × 10^−6^	3.22	17.23	0.305 ± 0.03	6.77 × 10^−8^ ± 0.00	0.414	
Top 10%	G1	9.47	1.70 × 10^−7^	2.49	17.29	0.324 ± 0.04	5.81 × 10^−9^ ± 0.00	0.409	0.79
	G2	2.06	0.39	10.59	17.12	0.068 ± 0.01	0.013 ± 0.01	0.432	0.21
	Gt	9.56	1.17 × 10^−6^	2.83	17.24	0.323 ± 0.05	3.95 × 10^−8^ ± 0.00	0.418	
Top 15%	G1	9.96	7.13 × 10^−7^	2.36	17.27	0.337 ± 0.06	2.41 × 10^−8^ ± 0.00	0.416	0.81
	G2	1.85	0.43	10.75	17.11	0.061 ± 0.01	0.014 ± 0.01	0.432	0.19
	Gt	9.90	6.31 × 10^−7^	2.85	17.22	0.330 ± 0.05	2.11 × 10^−8^ ± 0.00	0.425	
Top 20%	G1	10.03	5.53 × 10^−7^	2.63	17.25	0.335 ± 0.05	1.85 × 10^−8^ ± 0.00	0.423	0.82
	G2	1.73	0.51	10.80	17.11	0.057 ± 0.01	0.017 ± 0.01	0.433	0.18
	Gt	9.83	3.36 × 10^−7^	3.23	17.21	0.324 ± 0.04	1.11 × 10^−8^ ± 0.00	0.431	

Notes: $\sigma_{a}^{2}$: additive variance; $\sigma_{d}^{2}$: dominance variance; $\sigma_{p}^{2}$: permanent environmental variance; $\sigma_{e}^{2}$: residual variance; $h_{a}^{2}$: additive heritability; $h_{d}^{2}$ dominance heritability; r: repeatability.

**Table 4:** Estimates of CL variance components and genetic parameters based on the GBLUP–GA method under different top SNP proportions.

Prior Marker Information	Matrix	$\sigma_{a}^{2}$	$\sigma_{p}^{2}$	$\sigma_{e}^{2}$	$h_{a}^{2}$	r	τ
Top 5%	G	0.092	0.0001	0.908	0.092 ± 0.01	0.092	
	G1	0.108	5.87 × 10^−8^	0.922	0.105 ± 0.02	0.105	0.66
	G2	0.055	0.050	0.979	0.051 ± 0.01	0.097	0.34
	Gt	0.112	3.79 × 10^−6^	0.928	0.108 ± 0.03	0.108	
Top 10%	G1	0.112	4.87 × 10^−8^	0.922	0.108 ± 0.03	0.108	0.73
	G2	0.042	0.063	0.979	0.038 ± 0.01	0.097	0.27
	Gt	0.117	8.15 × 10^−6^	0.928	0.112 ± 0.02	0.112	
Top 15%	G1	0.116	4.79 × 10^−8^	0.925	0.111 ± 0.02	0.111	0.77
	G2	0.035	0.069	0.979	0.032 ± 0.01	0.096	0.23
	Gt	0.112	5.99 × 10^−6^	0.930	0.107 ± 0.02	0.107	
Top 20%	G1	0.120	4.71 × 10^−8^	0.927	0.114 ± 0.03	0.115	0.80
	G2	0.030	0.074	0.979	0.028 ± 0.01	0.092	0.20
	Gt	0.123	8.67 × 10^−6^	0.932	0.117 ± 0.03	0.105	

Notes: $\sigma_{a}^{2}$: additive variance; $\sigma_{p}^{2}$: permanent environmental variance; $\sigma_{e}^{2}$: residual variance; $h_{a}^{2}$: additive heritability; r: repeatability.

## Data Availability

The data presented in this study are available upon request from the corresponding author. The data are not publicly available because they are part of an ongoing research project led by the corresponding author that has not yet been finalized and are required for further in-depth analysis.

## Supplementary Materials

The following supporting information can be downloaded at: https://www.mdpi.com/article/10.3390/vetsci12100996/s1, Table S1: Impact of Different Prior Marker Information on the Accuracy of Genomic Prediction for CY; Table S2: Impact of Different Prior Marker Information on the Accuracy of Genomic Prediction for CD; Table S3: Impact of Different Prior Marker Information on the Accuracy of Genomic Prediction for BW; Table S4: Impact of Different Prior Marker Information on the Accuracy of Genomic Prediction for CL.

## Abbreviations

The following abbreviations are used in this manuscript:

IMCGs	Inner Mongolia cashmere goats
GWAS	Genome-Wide Association Study
SNP	single nucleotide polymorphism
CY	cashmere yield
CD	cashmere diameter
BW	body weight
CL	cashmere length
GEBV	Genomic Estimated Breeding Value
GS	Genomic Selection
