# Supplementary material for: Genomic Selection for Economic Traits in Inner Mongolia Cashmere Goats by Integrating GWAS Prior Information
Source: Vet Sci. 2025 Oct 15;12(10):996. doi: 10.3390/vetsci12100996 (PMC12568051; doi:10.3390/vetsci12100996)
Supplement: Supplementary file 1 [file vetsci-12-00996-s001.zip › vetsci-3828776-supplementary.pdf]

Table S1. Impact of Different Prior Marker Information on the Accuracy of Genomic Prediction for CY.

Method	Prior SNP Proportion	Accuracy	Accuracy Error	Improvement (%)
GBLUP∣GA	Original	0.7262	0.1000	
	5%	0.8156	0.0351	0.1231
	10%	0.8134	0.0361	0.1201
	15%	0.8081	0.0501	0.1128
	20%	0.8065	0.0545	0.1106

Table S2. Impact of Different Prior Marker Information on the Accuracy of Genomic Prediction for CD.

Method	Prior SNP Proportion	Accuracy	Accuracy Error	Improvement (%)
GBLUP∣GA	Original	0.6597	0.0960	
	5%	0.6083	0.1129	-0.0779
	10%	0.7293	0.1372	0.1055
	15%	0.8064	0.0909	0.2224
	20%	0.8074	0.0922	0.2239

Table S3. Impact of Different Prior Marker Information on the Accuracy of Genomic Prediction for BW.

Method	Prior SNP Proportion	Accuracy	Accuracy Error	Improvement (%)
GBLUP∣GA	Original	0.7099	0.0560	
	5%	0.8361	0.0102	0.1778
	10%	0.8213	0.0103	0.1569
	15%	0.8297	0.0105	0.1687
	20%	0.8212	0.0109	0.1568

Table S4. Impact of Different Prior Marker Information on the Accuracy of Genomic Prediction for CL.

Method	Prior SNP Proportion	Accuracy	Accuracy Error	Improvement (%)
GBLUP∣GA	Original	0.7271	0.0640	
	5%	0.7571	0.1205	0.0412
	10%	0.7504	0.1252	0.0320
	15%	0.7255	0.1202	-0.0022
	20%	0.7464	0.1292	0.0265
